# Fabrication and Deposition of Copper and Copper Oxide Nanoparticles by Laser Ablation in Open Air

**DOI:** 10.3390/nano10020300

**Published:** 2020-02-10

**Authors:** Mónica Fernández-Arias, Mohamed Boutinguiza, Jesús del Val, Antonio Riveiro, Daniel Rodríguez, Felipe Arias-González, Javier Gil, Juan Pou

**Affiliations:** 1 Applied Physics Department, University of Vigo, 36310 Vigo, Spain; mohamed@uvigo.es (M.B.); jesusdv@uvigo.es (J.d.V.); ariveiro@uvigo.es (A.R.); jpou@uvigo.es (J.P.); 2 Biomaterials, Biomechanics and Tissue Engineering Group, Materials Science and Metallurgical Engineering Department, UPC-Barcelona TECH, 08930 Barcelona, Spain; Daniel.rodriguez.rius@upc.edu; 3 School of Dentistry, Universitat Internacional de Catalunya, 08017 Barcelona, Spain; farias@uic.es (F.A.-G.); xavier.gil@uic.cat (J.G.)

**Keywords:** copper nanoparticles, laser ablation, antibacterial effects

## Abstract

The proximity of the “post-antibiotic era”, where infections and minor injuries could be a cause of death, there are urges to seek an alternative for the cure of infectious diseases. Copper nanoparticles and their huge potential as a bactericidal agent could be a solution. In this work, Cu and Cu oxide nanoparticles were synthesized by laser ablation in open air and in argon atmosphere using 532 and 1064 nm radiation generated by nanosecond and picosecond Nd:YVO_4_ lasers, respectively, to be directly deposited onto Ti substrates. Size, morphology, composition and the crystalline structure of the produced nanoparticles have been studied by the means of field emission scanning electron microscopy (FESEM), high resolution transmission electron microscopy (HRTEM), the energy dispersive spectroscopy of X-rays (EDS), selected area electron diffraction (SAED) and X-ray diffraction (XRD). The UV-VIS absorbance of the thin layer of nanoparticles was also measured, and the antibacterial capacity of the obtained deposits tested against *Staphylococcus aureus*. The obtained deposits consisted of porous coatings composed of copper and copper oxide nanoparticles interconnected to form chain-like aggregates. The use of the argon atmosphere contributed to reduce significantly the formation of Cu oxide species. The synthesized and deposited nanoparticles exhibited an inhibitory effect upon *S. aureus*.

## 1. Introduction

The decreasing effectiveness of antibiotics and other antimicrobial agents is a global concern. According to the World Health Organization (WHO), “the post-antibiotic era”, where no treatment for infections and minor injuries exists, is near [1]. Currently, antibiotic resistance kills an estimated 700,000 people each year, and some experts predict that the number could rise to 10 million by 2050, if efforts to curtail resistance or develop new antibiotics are not made [2].

*Staphylococcus aureus*, in addition to being related to a large number of infectious diseases, is one of the bacteria that presents a greater resistance to current commercial antibiotics [3]. Furthermore, several researchers have demonstrated the important role of this *S. aureus* in some oral infections such as peri-implantitis, which is considered the main cause of dental implant failure [4]. To promote an antimicrobial response from implants, some metallic antibacterial elements have been incorporated into implants’ surfaces and matrices [5,6]. Among these elements, noble metal and transition metal nanoparticles are attracting great interest due to their remarkable antibacterial properties.

Copper, besides the fact that it is potentially effective against different bacterial pathogens [7], is a trace element. That is, an element necessary for the proper functioning of the human body in amounts less than 100 mg per day, there being recommended a daily copper intake of around 1.4 mg for an adult weighing about 70 kg [8]. It works as an agent to help integrate iron, zinc and vitamin C, besides being essential for the brain and its neurotransmissions, energy production, and to regulate several hormonal processes. In addition, copper is part of a good number of enzymes, and is involved in tissue respiration [9,10]. 

In particular, the antibacterial activity of copper and its oxides when the size is reduced to nanoscale is of great interest because of a high surface to volume ratio, which allows doctors to kill the pathogens without affecting the healthy tissue that surrounds it [11]. But not only is the size behind these particular properties, but also shape of the particles, which depends upon the fabrication method. The technique of laser ablation in gaseous media used in the present study allows researchers to obtain nanoparticles with reduced average sizes. On the other hand, the absence of potentially toxic chemicals makes this technique a preferred choice for the preparation of nanoparticles. In other techniques, the use of surfactants or the use of chemical precursors produces the contamination of the nanoparticles with agents potentially toxic for human cells [12,13,14]. 

In previous works, silver nanoparticles were obtained by laser ablation in open air [15], and their antibacterial properties against *Lactobacillus salivarius* were then probed [16]. More recently, copper nanoparticles with different degrees of oxidation were also obtained by laser ablation in water and methanol, in order to demonstrate their cytocompatibility and bacteriological activity against *Aggregatibacter actinomycetemcomitans* [17]. In the present work, we report the synthesis and deposition of copper and copper oxide nanoparticles on cp Ti substrates in a one-step process by laser ablation. The process is carried out in the open air and in an argon atmosphere using two different laser sources. Results and formation process, including the influence of laser parameters, are discussed. Antibacterial activity against *Staphylococcus aureus* (a gram-positive aerobic bacteria normally associated with surgical wounds in orthopedic and dental patients) is also studied for being one of the most dangerous antibiotic-resistant bacteria.

## 2. Materials and Methods

### 2.1. Laser Ablation

**Figure 1:**
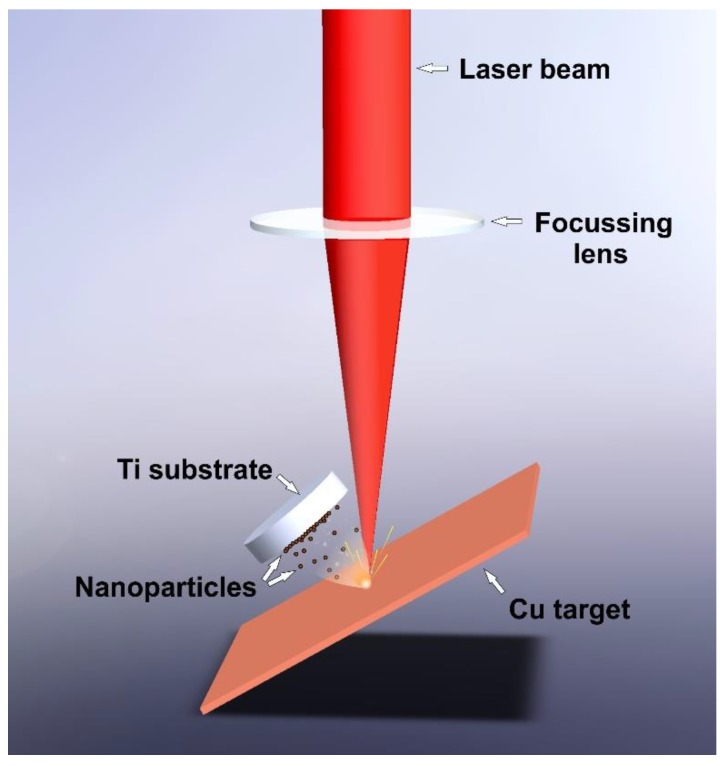
Laser ablation process.

A Copper foil with 99.99% of purity (Alfa Aesar) previously cleaned, was used as a laser ablation target. Titanium discs Grade 2 (Goodfellow Cambridge Limited, Huntingdon, UK) with 10 mm diameter and about 200 nm of average surface roughness were used as substrates to collect the ablated material. The target was set at a 30 degree angle with the horizontal plane, and the laser beam was focused on its upper surface. In each experiment, one Ti substrate was tilted to be placed almost parallel to the copper target, 10 mm away from the incident point of the laser beam, as detailed in Figure 1. 

Two different laser sources were used in the process. The first system was a diode-pumped Nd:YVO4 laser, providing pulses of 14 ns at a wavelength of 532 nm with 0.26 mJ of pulse energy. The second laser source was a Nd:YVO4 laser providing pulses of 800 ps at 1064 nm of wavelength and 0.03 mJ of pulse energy. The laser beam spot diameter on the target surface was estimated to be 132 μm, giving a fluence of 1.90 J/cm^2^ in the case of the Green-Nanosecond laser and 196 μm, giving a fluence of 0.09 J/cm^2^, for the IR–Picosecond laser. In all cases, the laser beam was kept in relative movement with respect to the target at 50 mm/s of scanning speed. The processing parameters used are listed in Table 1. 

To analyze in depth the formation process of nanoparticles and the influence of oxygen on this process, two different atmospheres were used with each laser source: In open air and in the argon environment by using an airtight chamber which kept the oxygen content below 50 ppm during the process. Note that all the assays were performed at atmospheric pressure (1 atm). Sample nomenclature with the corresponding assay conditions are listed in Table 2.

In order to compare the obtained samples, the same ablated mass (2 mg) for each condition was deposited on a titanium plate. Since the process parameters (shown in Table 1) and atmosphere changed, different processing time was required to obtain the same ablated mass as reported in Table 2. In this sense, it is noteworthy to mention that several previous assays were performed with each condition before the final preparation, in order to adjust carefully the required time to obtain the same mass. Additionally, in each final assay, the ablated mass was confirmed by weighing the targets before and after the ablation process (Δm ± 0.001 g) for a higher accuracy.

### 2.2. Sample Preparation and Characterization Technics

The morphology and composition of the nanoparticles on the titanium surface were characterized by using a Ga ion beam in the FEI Helios NanoLab 600 (FEI, Hillsboro, OR, USA) dual beam microscope. Scanning electron microscopy (SEM) micrographs and energy dispersive spectroscopy X-rays (EDS) on the sectioning layer of the coating were obtained by Focused Ion Beam (FIB).

The deposited nanoparticles on the titanium discs were observed by Field Emission Scanning Electron Microscopy (FESEM) with a JEOL JSM 6700F microscope (JEOL, Akishima, Japan). Carbon-coated copper microgrids were also used as substrates to collect the nanoparticles for a size and morphology analysis. Transmission Electron Microscopy (TEM) images were acquired with a JEOL JEM 1010 (JEOL, Akishima, Japan) microscope and the nanoparticle size distribution was derived from a histogram obtained by measuring the diameter of about 300 particles.

Nanoparticles were also deposited on carbon-free copper microgrids in order to accomplish a crystallographic characterization. High-Resolution Transmission Electron Microscopy (HRTEM) and Selected Area Electron Diffraction (SAED) images were acquired with a JEOL JEM 2010F (JEOL, Akishima, Japan) high-resolution transmission electron microscope, equipped with a slow digital camera scan, using a 200 kV accelerating voltage. Identification of phases was achieved by comparing the measured distances with the diffraction patterns from the ICDD (JCPDS) database.

In order to corroborate the composition of the obtained nanoparticles, X-ray diffraction (XRD) analysis was carried out by means of a PANanalytical X’Pert Pro X-ray diffractometer using monochromated Cu-Kα radiation (wavelength 1.54 Å) over the 20–100° 2θ range with a step size of 0.02°. To facilitate the phase identification, the nanoparticles were deposited on a zero-background holder. The diffraction peaks of each sample were compared with the reference pattern of pure copper and different copper oxides from the ICDD (JCPDS) database.

The same ablation process was repeated by using a piece of glass to collect the nanoparticles, with the purpose of studying the optical properties. The ultraviolet to visible (UV/VIS) absorption spectrum of the copper nanoparticles’ layer was measured in the range from 280 to 800 nm, using a Hewlett Packard HP 8452 diode array spectrophotometer.

Finally, three replicas (titanium discs with NPs) of each condition were submerged in 25 mL of ultrapure, deionized water in order to study the influence of copper ions in the bactericidal process. From each replica, 1.5 mL were laid away regularly during the first 21 days to be centrifuged. The extracted volume (1.5 mL) was gently replaced with fresh, deionized water. Afterwards, possible nanoparticles were separated from the solutions by using an Eppendorf miniSpin centrifuge at 13,400 rpm for 30 min at room temperature. After centrifuging, only 1 mL from the upper surface was taken, leaving the heaviest matter (NPs) in the bottom. In this regard, in order to ensure that only the ions in suspension are measured, UV-VIS spectroscopy was also used before analyzing. Finally, the ions content in the solutions was measured by Inductively Coupled Plasma Optical Emission Spectrometry (ICP-OES) with an Optima 4300 DV (Perkin Elmer, Waltham, MA, USA).

### 2.3. Antimicrobial Activity

The antibacterial activity of the treated samples was studied with *Staphylococcus aureus* (CECT (Colección Española de Cultivos Tipo) 435, Valencia, Spain) cultured in BHI broth (Scharlab SL, Sentmenat, Spain). A bacteria inoculum was incubated for 24 h at 37 °C before the assay. 

The optical density for the bacterial suspension was adjusted to 0.2 ± 0.01 at 600 nm, equivalent to 1 × 10^8^ colony-forming units (CFU)/mL. 

The control and treated samples were immersed in ethanol and distilled water for 15 min each, and put into a 24-multiwell plate (Nunc, Rochester, NY, USA) with 1 mL bacterial suspension at 37 °C for 2 h. Afterwards, the samples were washed thrice with phosphate buffered solution (PBS) to wash off the nonadherent bacteria. The adhered bacteria were collected, sonicating the samples in 1 mL sterile PBS for 5 min. The PBS was serially diluted, and the diluted bacterial suspensions were seeded onto agar plates supplemented with BHI medium. The agar plates were then incubated at 37 °C for 24 h, and the CFUs were counted.

### 2.4. Statistical Analysis

Data collected from the antibacterial assay were statistically analyzed. All quantitative values were presented as mean ± standard deviation (SD). All experiments were performed using three replicates. Tukey’s test was applied for comparing the mean of each group with the mean of the control group, and the means of all groups in pairs with a level of significance *p*-value < 0.05.

## 3. Results and Discussion

The characteristics and properties of the obtained nanoparticles were investigated, and the results analyzed concerning the influence of different parameters in the ablation process.

### 3.1. Ablation Rate

In order to ensure that the same quantity of ablated material was produced for each condition, the ablation time was carefully adjusted, being required to be at a different duration for each processing condition (see Table 2). This information gives us an idea about the efficiency of the process depending on the laser source and the atmosphere used, and also makes it possible to analyze the influence of time on the resultant nanoparticles. 

As detailed in Table 2, the Green-Nanosecond laser requires more than twice as long as the IR–Picosecond to ablate the same amount of material from the copper plate surface. These results evidence the influence of laser parameters on the process. According to previous works, the ablation rate of pure metals is higher at low fluence due to the plasma shielding effect [18]. During the laser–matter interaction, material from the surface of the target is ejected and the plasma plume appears. This plasma reaches temperatures of tens of thousands Kelvin in the ablation process, but it is even higher in case of high fluences. When this occurs, as a result of the temperature increase, the plasma expands, and a protective shield on the upper surface of the material appears [19]. In addition, the absorption coefficient of the copper is higher for the 532 nm (green) than for the 1064 nm (invisible) wavelength, leading to a self-absorption process, where part of the incoming laser radiation is absorbed by the ablated material [20]. In the aforementioned processes (plasma shielding and self-absorption), the ablated material absorbs part of the incoming laser radiation, and consequently, the energy which reaches the surface is attenuated. Both phenomena have a great influence on the plasma development, and consequently on the amount of the ablated material.

Although laser parameters such as laser fluence, wavelength and pulse duration are important for controlling the process, the ablation environment determines to a large extent the laser–material interaction [21,22]. In this sense, the ablation process seems to be more productive in open air than in argon. This is likely due to the presence of oxygen and the high reactivity of copper. With about 20% of the background, air seems to contribute to increase the ablation efficiency by means of an exothermal reaction. As it was addressed by other authors, oxygen molecules dissociated in the process due to the high temperatures, and oxidized the surface of the target favoring the reaction and the heating of the material [18]. In addition, the atomic weight of the gaseous media has a great influence upon the plasma development. In this sense, Ar with a higher atomic weight than air conduces to more confinement of the plasma plume and a slow expansion. 

### 3.2. Physicochemical Characterization of the Film

**Figure 2:**
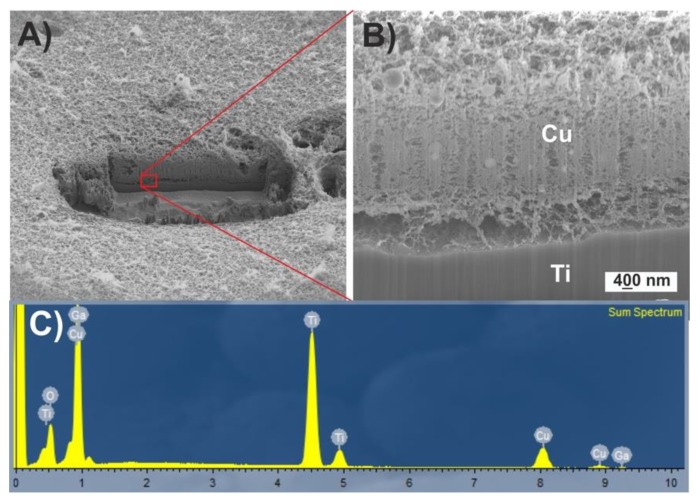
(**A**) Scanning electron microscopy (SEM) micrograph of the nanoparticles coating on the titanium disc, (**B**) SEM micrograph of the Focused Ion Beam (FIB)-prepared cross-section, (**C**) Sum spectrum obtained from the cross-section.

FIB-prepared cross-section shows the arrangement of the deposited nanoparticles on the titanium surface (Figure 2A,B). SEM micrographs show micrometer nanoparticles immersed inside a spongy coating of small nanoparticles. The adhesion between titanium and nanoparticles seems to be weak due to the small contact surface between them.

EDS performed confirms the composition of the coating.

### 3.3. Characterization of the Obtained Nanoparticles

#### 3.3.1. Size and Morphology

**Figure 3:**
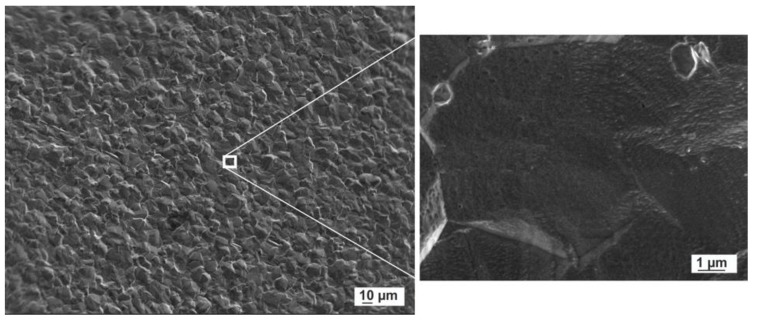
Field emission scanning electron microscopy (FESEM) micrographs of titanium disc surface used as substrate.

Several images were recorded and used to perform an analysis of morphology and size distribution. As reference, a FESEM image of the Titanium substrate surface was also acquired (see Figure 3). 

**Figure 4:**
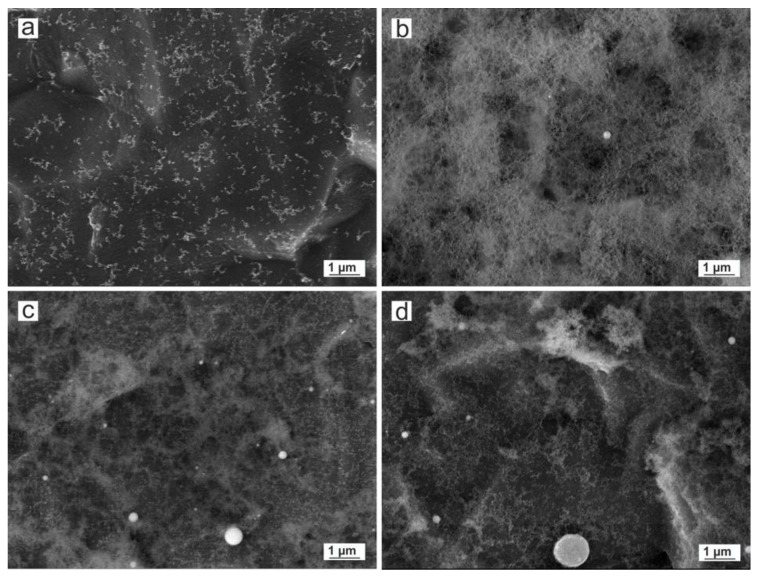
FESEM micrographs of Cu nanoparticles from samples a, b, c and d obtained by laser ablation using (**a**) Green-Nanosecond laser in air, (**b**) Green-Nanosecond laser in argon, (**c**) IR-Picosecond laser in air, (**d**) IR-Picosecond laser in Argon, as reported in Table 2.

Figure 4 shows the aspect of the obtained copper nanoparticles anchored on the titanium surface at low magnification. 

Note that large particles can be observed on the titanium surface. This result is a common consequence of laser ablation due to the formation mechanism of nanoparticles. When laser pulses of high intensity strike on the copper target, the temperature of the interaction zone is increased up to its boiling point, leading to an explosive boiling, where nanodroplets together with ionized matter (ions, clusters, free atoms) are ejected from the target to the substrate surface. These nanodroplets, can form spherical micrometric and submicrometric particles and solidify on the substrate, while the ionized matter nucleates and grows. The EDS analysis confirms that even the micrometric particles are copper or copper oxide.

**Figure 5:**
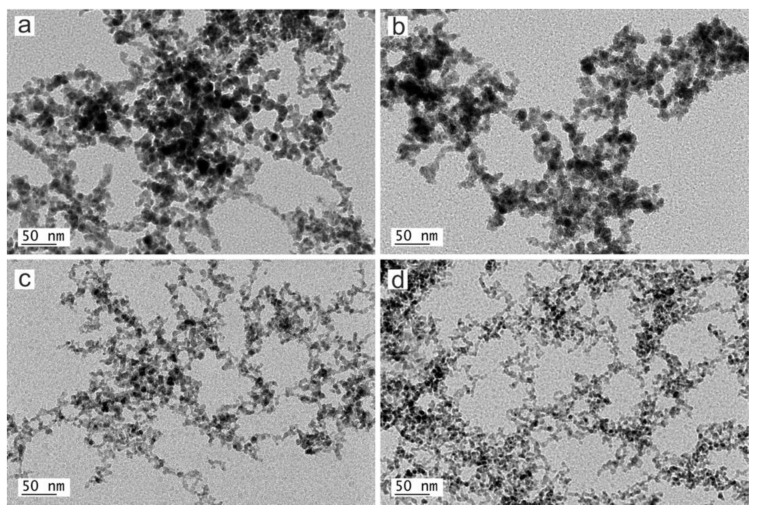
Transmission electron microscopy (TEM) micrographs of Cu nanoparticles from samples a, b, c and d obtained by laser ablation using (**a**) Green-Nanosecond laser in air, (**b**) Green-Nanosecond laser in argon, (**c**) IR-Picosecond laser in air, (**d**) IR-Picosecond laser in argon, as reported in Table 2.

TEM micrographs of the obtained copper nanoparticles are shown in Figure 5.

As can be observed at higher magnification, copper nanoparticles obtained by laser ablation exhibit a rounded shape with a high tendency to agglomerate, forming chain-like structures. This is because of the metallic nature of the starting material and the formation mechanism of the nanoparticles. Concurrently with the nucleation and growth process of nanoparticles, the absorbed radiation increases the temperature of the already formed NPs above the melting point, melting and joining with others by coalescence, leading to the formation of chain-like structures. 

**Figure 6:**
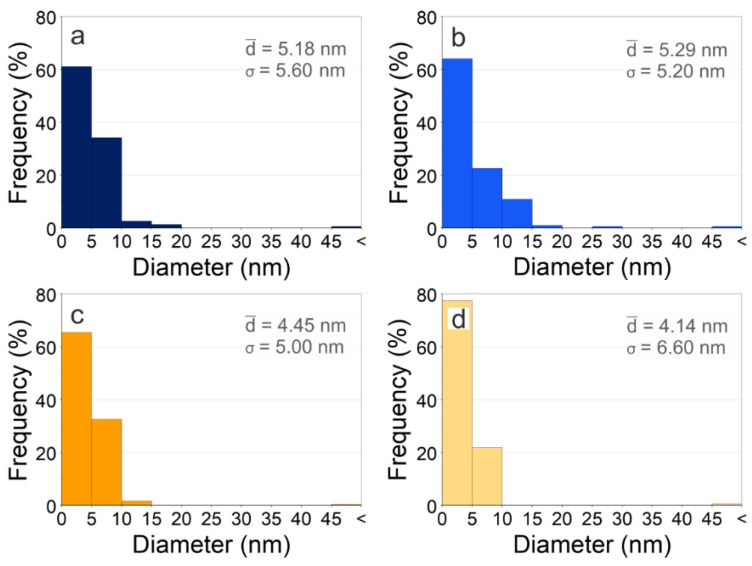
Histograms of size distribution from Cu nanoparticles obtained by laser ablation with (**a**) Green-Nanosecond laser in air, (**b**) Green-Nanosecond laser in argon, (**c**) IR-Picosecond laser in air, (**d**) IR-Picosecond laser in argon.

The represented histograms (see Figure 6) were obtained from several representative TEM images by measuring the diameter of about 300 particles of each sample. 

Close inspection of Figure 6 reveals that the sizes of the obtained nanoparticles are deeply determined by the nucleation time and the growth time of nuclei. In this growth kinetics, parameters such as fluence, wavelength and the repetition rate of the laser source, but also the environmental conditions, are critical factors [21,23].

As can be seen, copper nanoparticles obtained by the IR–Picosecond laser in argon present the lowest average size and the highest dispersion. On one hand, the fraction of particles of small size (between 0 and 5 nm) is greater when the laser ablation takes place in argon. These results bring to light the great influence of the atmosphere on the ablation process. In argon, the absorption of the laser beam by the plasma is higher than in air, and consequently, less of the sample is vaporized [21]. On the other hand, the broad size dispersion is due to the presence of large particles from the material ejection during the laser ablation process. These micrometric particles occur more frequently with the IR-Picosecond laser, because, although the power is similar, the pulse energy is concentrated in shorter pulses.

#### 3.3.2. Composition and Crystallography

##### HRTEM, FFT and SAED

**Figure 7:**
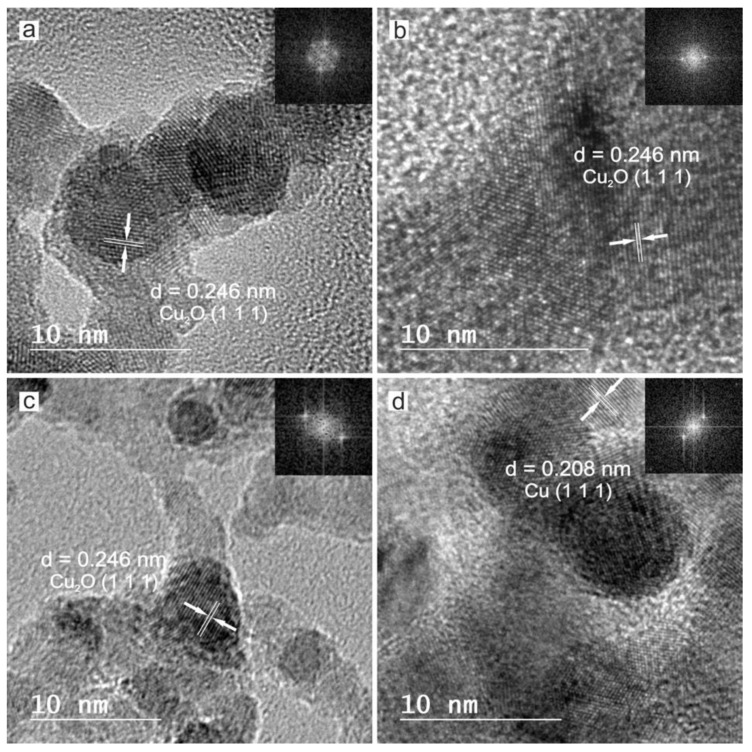
This is high resolution transmission electron microscopy (HRTEM) images of copper crystalline nanoparticles obtained by laser ablation with (**a**) Green-Nanosecond laser in air, (**b**) Green-Nanosecond laser in argon, (**c**) IR-Picosecond laser in air, (**d**) IR-Picosecond laser in argon. Measured interplanar distances and the corresponding Fast Fourier Transform (FFT) of single nanoparticles inset.

A high resolution transmission electron microscope was used to reveal the crystalline structure of the obtained nanoparticles. All of the particles obtained, even the smallest ones, are crystalline. This aspect can be observed in Figure 7, showing HRTEM of polycrystalline nanoparticles with clear lattice fringes and their corresponding Fast Fourier Transform (FFT) of the characteristic crystal as insets. 

**Figure 8:**
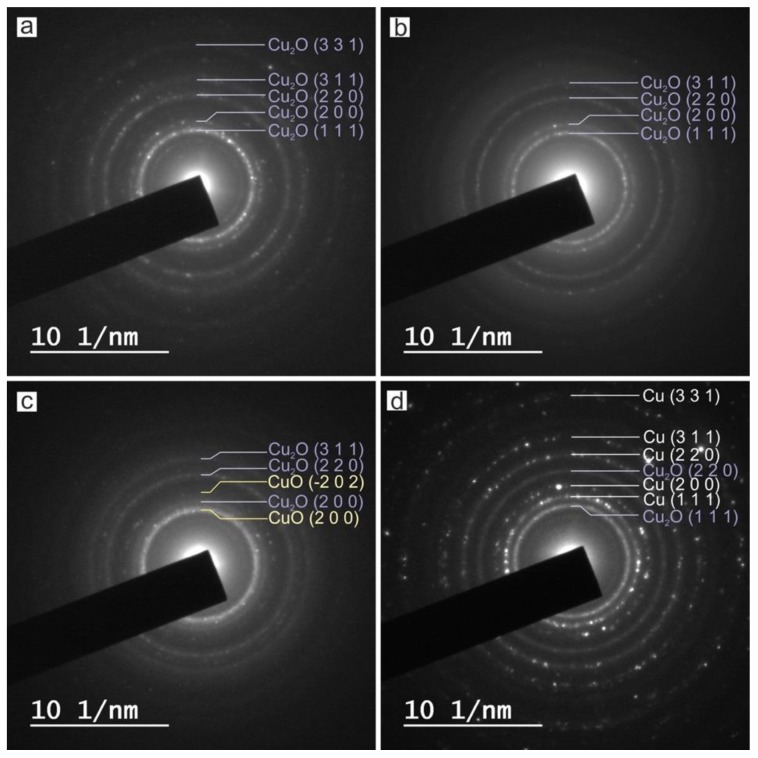
Selected area electron diffraction (SAED) pattern obtained over a group of copper nanoparticles obtained by laser ablation with (**a**) Green-Nanosecond laser in air, (**b**) Green-Nanosecond laser in argon, (**c**) IR Picosecond laser in air, (**d**) IR-Picosecond laser in argon.

In order to elucidate the crystalline phases of each sample, SAED was performed on several groups of particles, as shown in Figure 8. Identification of phases was achieved by comparing the measured distances from SAED with the diffraction patterns from the ICDD (JCPDS) database.

The measured interplanar distances from Figure 8 are listed in Table 3.

According to the data collected in Table 3, the main reflections of each sample correspond with diffraction patterns of metallic copper and different oxidation states of copper from the ICDD (JCPDS) database. The main reflections hkl of Cu-NPs obtained by laser ablation using the Green-Nanosecond laser in air (sample a) and argon (sample b), can be indexed on the cubic crystal lattice of Cu_2_O (JCPDS-ICDD ref. 005-0667), while the Cu-NPs obtained by laser ablation using the IR-Picosecond laser in air (sample c) corresponds with a combination of Cu_2_O and CuO (JCPDS-ICDD ref. 041-0254), and a combination of Cu_2_O and Cu (JCPDS-ICDD ref. 004-0836) if the IR-Picosecond laser is used in argon (sample d). 

##### XRD

**Figure 9:**
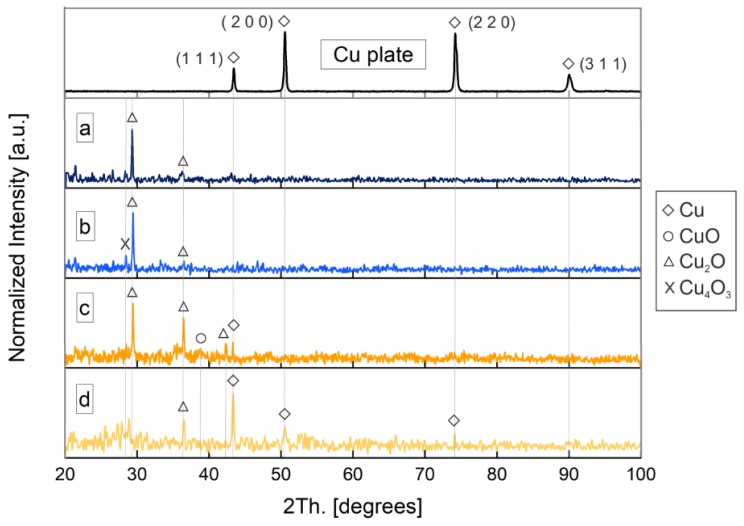
X-ray diffractometry (XRD) of Cu nanoparticles obtained with a wavelength of 532 nm by Los Alamos National Laboratory (LASL) with (**a**) the Green-Nanosecond laser in air, (**b**) Green-Nanosecond laser in argon, (**c**) an IR-Picosecond laser in air, (**d**) and the IR-Picosecond laser in argon. Gray lines represent the position of the representative diffraction peaks.

To elucidate the crystalline phases, X-ray diffractometry (XRD) was performed on the obtained nanoparticles and the precursor copper plate. The corresponding patterns of the samples are depicted in Figure 9. 

As can be clearly seen, the diffraction pattern of the target corresponds to a cubic crystal system with characteristic diffraction peaks (111), (200), (220) and (311) at 2θ values of 43.3°, 50.4°, 74.1° and 89.9°, respectively, according to JCPDS-ICDD ref. 004-0836. The elemental nature of the obtained Cu NPs was confirmed through XRD analysis. Samples obtained by laser ablation with the Green-Nanosecond laser (samples a and b) are mainly composed of Cu_2_O. Although there is the presence of an intensity peak at 28.5° corresponding to Cu_4_O_3_ (JCPDS-ICDD ref. 003-0879) in sample b, it suggests that copper nanoparticles obtained in argon are less oxidized. On its behalf, Cu NPs obtained by laser ablation with the IR–Picosecond laser in air (sample c) corresponds with Cu_2_O and a low presence of Cu and CuO, while in argon (sample d) NPs correspond mainly with metallic copper.

It is noteworthy to mention, that although the samples obtained in an inert atmosphere present a lower oxidation degree, the insignificant difference between samples a and b compared to the samples obtained when the IR–Picosecond laser, is probably due to the different mechanisms of NPs formation. When the Picosecond laser is used, ablated material is directly ejected from the surface target as a result of the direct sublimation. On the contrary, in the Nanosecond regime, the ablated material is melted and vaporized, growing in the plasma as a consequence of the interaction with the ambient gas, and subsequently by coalescence on the substrate. This thermal process favors the oxidation of the final NPs.

Note that although the results from XRD are in good agreement with those from SAED, the first one gives us more significant information. This is because, while in SAED the diffraction is performed on a group of particles, the scanned area in XRD is much larger. Furthermore, the corresponding peak positions of Cu and Cu_2_O among samples match, but the intensities are quite different. 

As reported by Ingham et al. [24], these results are common in thin films, and reveal a preferential orientation of each sample.

#### 3.3.3. UV-VIS Absorption

**Figure 10:**
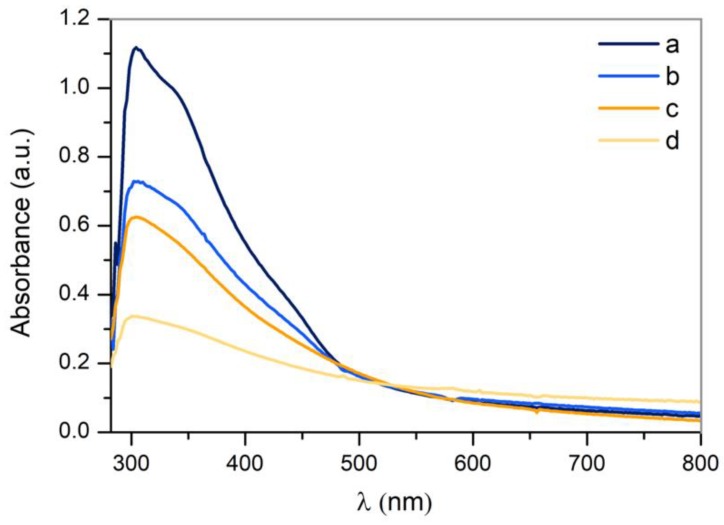
UV-VIS spectrum of Cu nanoparticles obtained by laser ablation using the Green-Nanosecond laser in (**a**) air, (**b**) argon and using the IR-Picosecond laser in (**c**) air, (**d**) Argon.

In order to study the optical properties of the obtained nanoparticles, the absorbance of each sample was measured in the range of 280–800 nm by UV-VIS spectroscopy (Figure 10). 

The mean peak, present in all samples at approximately 300 nm, is characteristic of surface plasmon resonance (SPR), a feature of CuO nanoparticles [25,26], although curves corresponding to samples a and b seem to be a convolution of two peaks. The mean peak corresponds to CuO and there is another at 350 nm, which corresponds with Cu_2_O [25]. This hypothesis is supported by the results previously obtained by means of the crystallographic analysis. It is noteworthy to mention that SPR is a resonance condition, and occurs when the frequency of the incident light matches with the surface electron frequency of NPs [27]. As it was addressed by previous authors, this effect may not be observed in all nanoparticles, since only nanoparticles larger than 20 nm present SPR [28]. Furthermore, the reactive nature of copper extensively used in catalysis may be responsible for the subsequent oxidation by contact with atmospheric oxygen [29]. On the other hand, the SPR is associated to the nanoparticles’ shape, size and surrounding medium. In this sense, the presence of a single surface plasmon peak implies that they are spherical [30], and the broadening of the absorbance peak is characteristic of a wide size distribution [31], which is in agreement with the TEM analysis. 

#### 3.3.4. Copper Ion Release

In order to study the ion release kinetics from the deposited nanoparticles, the copper ions content in water was measured during the first 21 days after ablation.

**Figure 11:**
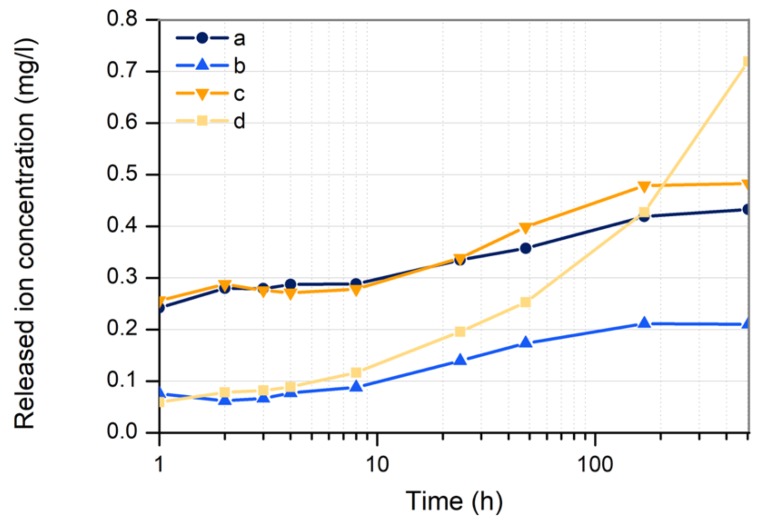
Kinetics of copper ion release from the immobilized copper nanoparticles on Ti plates.

As can be seen in Figure 11, nanoparticles deposited by laser ablation in open air (samples a and c) show a higher rate of ion release than Cu-NPs deposited in argon (samples b and d), being almost four times higher in the first 8 h. This suggests that although the mass of copper nanoparticles deposited in each sample is the same (2 mg), the composition of the environment determines to a large extend the laser–matter interaction. Thus, when argon is used as a gaseous environment, nanoparticles could be deposited onto the surface of the substrate being embedded. This would reduce the nanoparticle surface in contact with the release medium. On the contrary, Cu nanoparticles obtained in open air would lead to a more superficial coating. 

Note that after the first 8 h, Cu nanoparticles obtained in Argon with the IR–Picosecond increase the ions’ release ratio. After 7 days (168 h), samples a, b and c seem to become stable.

### 3.4. Analysis of Antimicrobial Activity

**Figure 12:**
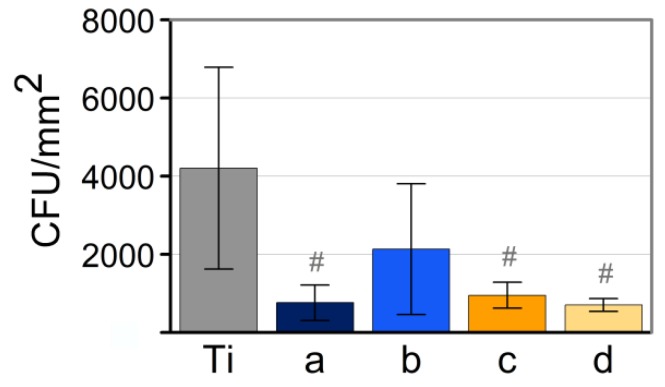
Bacterial adhesion on Ti with and without copper nanoparticles after 24 h of culture. Significant difference of each group with the control group (# *p*-value < 0.05).

In order to assess the bactericidal activity of the obtained NPs, the analysis was performed with a Gram-positive bacteria, *Staphylococcus aureus*. The obtained relative absorbance of each sample with the corresponding error is shown in Figure 12. Taking into account that the higher the absorbance, the more the bacterial growth, these values were compared with a positive control (titanium discs with bacteria without NPs). As is shown, after 24 h, the bacterial growth values are noteworthily reduced by all samples. Copper nanoparticles obtained by laser ablation in argon by using the IR–Picosecond laser (sample d), exhibit the highest bactericidal capacity. 

The statistical analysis performed shows differences between control and samples a, c and d. On the contrary, no statistically significant differences between samples a, c and d was observed. The notable dispersion in the results is consistent with the wide range of surface roughness on the titanium discs. In this sense, several authors have addressed the high impact of titanium surface topography on the bacterial adhesion [32,33].

Note that copper nanoparticles with the highest bactericidal effect (sample d) correspond to those that underwent a lower oxidation degree and smaller mean size with a high size dispersion. On one hand, previous work showed that a high oxidation state promotes the bactericidal activity but in conditions of similar oxidation states, particle size seems to be a decisive parameter. 

The smaller the size, the greater the antibacterial capacity. This is consistent with previous works, and demonstrates that NPs with a large surface-to volume ratio provide more antibacterial activity [7]. On the other hand, although previous works state a direct relationship between the bactericidal activity of copper nanoparticles and their ions’ release [6], our results provide compelling evidence that ions’ release is not crucial compared to other parameters, such as particle size or oxidation state. In general terms, our results are consistent with previous studies [7,34], where copper exhibits excellent antibacterial properties when tested on *S. aureus*.

## 4. Conclusions

Crystalline copper and copper oxide nanoparticles have been obtained by means of a laser ablation technique in gaseous media, using two different nanosecond Nd:YVO4 lasers working at 532 nm and 1064 nm of wavelength, and without any chemical reagent or contamination. 

The laser ablation ratio is higher with the IR–Picosecond laser than with the Green–Nanosecond. The presence of oxygen contributes to the process efficiency, obtaining more ablated material when laser ablation is carried out in open air than in argon. Nanoparticles obtained in argon do present in general terms a lower degree of oxidation than those obtained in air.

The antibacterial assay proved the strong antibacterial activity of the obtained copper nanoparticles against *S. aureus*. The best inhibitory effects are provided by copper nanoparticles obtained by laser ablation in argon with 1064 nm of wavelength. These results confirm the influence of size, crystallographic structure and oxidation state in the bactericidal effects of copper nanoparticles.

## Figures and Tables

**Table 1:** Processing parameters.

Laser Source	Wavelength (nm)	Pulse Length (ns)	Pulse Frequency (kHz)	Pulse Energy (mJ)	Scanning Speed (mm/s)
Green-Nanosecond	532	14	20	0.26	50
IR-Picosecond	1064	0.8	200	0.03	50

**Table 2:** Samples produced and analyzed.

Sample	Laser Source	Atmosphere	Time (min)
a	Green-Nanosecond	air	5
b	Green-Nanosecond	argon	7
c	IR-Picosecond	air	2
d	IR-Picosecond	argon	3

**Table 3:** Lattice spacing measured in nm from SAED and the corresponding diffraction patterns from the ICDD database of metallic Copper, CuO and Cu_2_O.

a	b	c	d	Cu (hkl)	CuO (hkl)	Cu_2_O (hkl)
0.247	0.246		0.248			0.247 (111)
		0.234			0.231 (200)	
			0.208	0.209 (111)		
0.214	0.216	0.213				0.214 (200)
			0.183	0.181 (200)		
		0.187			0.187 (−202)	
0.152	0.151	0.151	0.151		0.150 (−113)	0.151 (220)
0.129	0.132	0.129	0.128	0.128 (220)		0.129 (311)
			0.109	0.109 (311)		
0.098			0.083	0.083 (331)		0.098 (331)

## References

[1] Reardon S. (2014). WHO warns against ‘post-antibiotic’ era. Nature.

[2] Willyard C. (2017). Drug-resistant bacteria ranked. Nature.

[3] Ondusko D.S., Nolt D. (2018). Staphylococcus aureus. Pediatr. Rev..

[4] Goudouri O., Kontonasaki E., Lohbauer U., Boccaccini A.R. (2014). Antibacterial properties of metal and metalloid ions in chronic periodontitis and peri-implantitis therapy. Acta Biomater..

[5] Massa M.A., Covarrubias C., Bittner M., Fuentevilla I.A., Capetillo P., von Marttens A., Carvajal J.C. (2014). Synthesis of new antibacterial composite coating for titanium based on highly ordered nanoporous silica and silver nanoparticles. Mater. Sci. Eng. C.

[6] Palza H., Quijada R., Delgado K. (2015). Antimicrobial polymer composites with copper micro- and nanoparticles: Effect of particle size and polymer matrix. J. Bioact. Compat. Polym..

[7] Khalid H., Shamaila S., Zafar N., Sharif R., Nazir J., Rafique M., Ghani S., Saba H. (2016). Antibacterial Behavior of Laser-Ablated Copper Nanoparticles. Acta Metall. Sin. Engl. Lett..

[8] World Health Organization (1996). Trace Elements in Human Nutrition and Health.

[9] Karlin K.D. (1993). Metalloenzymes, Structural Motifs, and Inorganic Models. Science.

[10] Skalnaya M.G., Skalny A.V. (2018). Essential Trace Elements in Human Health: A Physician’S View.

[11] Vimbela G.V., Ngo S.M., Fraze C., Yang L., Stout D.A. (2017). Antibacterial properties and toxicity from metallic nanomaterials. Int. J. Nanomed..

[12] Wagener P., Jakobi J., Rehbock C., Chakravadhanula V.S.K., Thede C., Wiedwald U., Bartsch M., Kienle L., Barcikowski S. (2016). Solvent-surface interactions control the phase structure in laser-generated iron-gold core-shell nanoparticles. Sci. Rep..

[13] Boutinguiza M., Pou J., Lusquiños F., Comesaña R., Riveiro A. (2011). Laser-assisted production of tricalcium phosphate nanoparticles from biological and synthetic hydroxyapatite in aqueous medium. Appl. Surf. Sci..

[14] Boutinguiza M., Lusquiños F., Riveiro A., Comesaña R., Pou J. (2009). Hydroxylapatite nanoparticles obtained by fiber laser-induced fracture. Appl. Surf. Sci..

[15] Boutinguiza M., Comesaña R., Lusquiños F., Riveiro A., del Val J., Pou J. (2014). Production of silver nanoparticles by laser ablation in open air. Appl. Surf. Sci..

[16] Boutinguiza M., Fernández-Arias M., del Val J., Buxadera-Palomero J., Rodríguez D., Lusquiños F., Gil F.J., Pou J. (2018). Synthesis and deposition of silver nanoparticles on cp Ti by laser ablation in open air for antibacterial effect in dental implants. Mater. Lett..

[17] Fernández-Arias M., Boutinguiza M., del Val J., Covarrubias C., Bastias F., Gómez L., Maureira M., Arias-González F., Riveiro A., Pou J. (2019). Copper nanoparticles obtained by laser ablation in liquids as bactericidal agent for dental applications. Appl. Surf. Sci..

[18] Vadillo J.M., Fernández J.M., Rodríguez C., Laserna J.J. (1999). Effect of Plasma Shielding on Laser Ablation Rate of Pure Metals at Reduced Pressure. Surf. Interface Anal..

[19] Gottfried J.L. (2014). Influence of exothermic chemical reactions on laser-induced shock waves. Phys. Chem. Chem. Phys..

[20] Maina M., Okamoto Y., Inoue R., Nakashiba S., Okada A., Sakagawa T. (2018). Influence of Surface State in Micro-Welding of Copper by Nd:YAG Laser. Appl. Sci..

[21] Hermann J., Gerhard C., Axente E., Dutouquet C. (2014). Comparative investigation of laser ablation plumes in air and argon by analysis of spectral line shapes: Insights on calibration-free laser-induced breakdown spectroscopy. Spectrochim. Acta Part B At. Spectrosc..

[22] Hamad A.H. (2016). Laser Ablation in Different Environments and Generation of Nanoparticles Generation. Appl. Laser Ablation Thin Film Depos. Nanomater. Synth. Surf. Modif..

[23] Yang G.W. (2007). Laser ablation in liquids: Applications in the synthesis of nanocrystals. Prog. Mater. Sci..

[24] Ingham B. (2014). X-Ray Diffraction for Characterizing Metallic Films. Metallic Films for Electronic, Optical and Magnetic Applications.

[25] Raghav R., Aggarwal P., Srivastava S. (2016). Tailoring oxides of copper-Cu_2_O and CuO nanoparticles and evaluation of organic dyes degradation. AIP Conf. Proc..

[26] Zhu J., Li D., Chen H., Yang X., Lu L., Wang X. (2004). Highly dispersed CuO nanoparticles prepared by a novel quick-precipitation method. Mater. Lett..

[27] Khashan K.S., Jabir M.S., Abdulameer F.A. (2018). Carbon Nanoparticles decorated with cupric oxide Nanoparticles prepared by laser ablation in liquid as an antibacterial therapeutic agent. Mater. Res. Express.

[28] Díaz-Visurraga J., Daza C., Pozo C., Becerra A., von Plessing C., García A. (2012). Study on antibacterial alginate-stabilized copper nanoparticles by FT-IR and 2D-IR correlation spectroscopy. Int. J. Nanomed..

[29] Goncharova D., Lapin I., Svetlichnyi V. (2019). Structure and optical properties of nanoparticles obtained by pulsed laser ablation of copper in gases. J. Phys. Conf. Ser..

[30] Mafune F., Kohno J., Takeda Y., Kondow T. (2000). Formation and Size Control of Silver Nanoparticles by Laser Ablation in Aqueous Solution. J. Phys. Chem. B.

[31] Dadras S., Torkamany M.J., Jafarkhani P. (2012). Analysis and optimization of silver nanoparticles laser synthesis with emission spectroscopy of induced plasma. J. Nanosci. Nanotechnol..

[32] Truong V.K., Lapovok R., Estrin Y.S., Rundell S., Wang J.Y., Fluke C.J., Crawford R.J., Ivanova E.P. (2010). The influence of nano-scale surface roughness on bacterial adhesion to ultrafine-grained titanium. Biomaterials.

[33] Schubert A., Wassmann T., Holtappels M., Kurbad O., Krohn S., Bürgers R. (2019). Predictability of Microbial Adhesion to Dental Materials by Roughness Parameters. Coatings.

[34] Drelich J., Li B., Bowen P., Hwang J., Mills O., Hoffman D. (2011). Vermiculite decorated with copper nanoparticles: Novel antibacterial hybrid material. Appl. Surf. Sci..

